# Impact of multimodal health education and preoperative ocular fixation training on psychological stress and postoperative complications in patients undergoing pterygium surgery

**DOI:** 10.1515/med-2026-1480

**Published:** 2026-07-07

**Authors:** Shi-Jie Zhang

**Affiliations:** Department of Ophthalmology Center, Xining No. 1 People’s Hospital, Xining, Qinghai, P.R. China

**Keywords:** complications, multimodal health education, ocular fixation training, psychological stress, pterygium, surgery

## Abstract

**Objectives:**

This study evaluated the effects of multimodal health education combined with preoperative ocular fixation training on patients undergoing pterygium surgery.

**Methods:**

A cohort of 88 patients who underwent pterygium surgery at Xining First People’s Hospital from April 2025 to September 2025 were selected and randomly assigned to a control group (n=44) and an intervention group (n=44) using a random number table. The control group received routine high-quality nursing, and the intervention group received multimodal health education and preoperative ocular fixation training in addition to routine care. Disease uncertainty, psychological stress scores, and postoperative complications were compared between the two groups.

**Results:**

Following the intervention, patients in the intervention group had significantly lower Mishel Uncertainty in Illness Scale (MUIS) scores compared with the control group (p<0.05). Psychological resilience scores were significantly higher in the intervention group (p<0.05). The incidence rates of eye pain (11.36 %), infection (0), and dry eye (0) in the intervention group were lower than those observed in the control group (79.55 , 13.64, and 15.91 %, respectively) (p<0.05).

**Conclusions:**

Multimodal health education combined with preoperative ocular fixation training markedly reduced psychological stress and illness uncertainty in patients undergoing pterygium surgery and was associated with lower postoperative complication rates.

## Introduction

Pterygium progressively extends onto the cornea following onset, inducing astigmatism and potentially restricting ocular movement, which may result in binocular visual dysfunction. Surgical removal of proliferative tissue represents an essential intervention to control disease progression [1]. However, psychological stress frequently develops among patients due to uncertainty about surgical procedures and prognosis, which may reduce intraoperative cooperation and increase postoperative discomfort, thereby affecting overall recovery quality [2]. Routine high-quality nursing lacks targeted and multidimensional psychological support, and its preoperative health education remains relatively single-dimensional, limiting its ability to comprehensively address the complex needs of surgical patients [3].

Multimodal health education, which integrates verbal and written explanations with visual materials, enhances patients’ understanding and sense of control over the surgery from cognitive and emotional perspectives [4]. Preoperative ocular fixation training familiarizes patients with procedural requirements, improves cooperation during local anesthesia, and reduces surgical risks associated with involuntary ocular movement. The combined implementation of these interventions stabilizes emotional state and intraoperative eye positioning, alleviates psychological stress, minimizes tissue injury, and reduces related complications, collectively supporting surgical safety and postoperative recovery quality [5].

Based on this rationale, this study evaluated the effectiveness of multimodal health education combined with preoperative ocular fixation training in patients undergoing pterygium surgery.

## Data and methods

### General information

Patients who underwent pterygium surgery at Xining First People’s Hospital from April 2025 to September 2025 were screened for eligibility.

Inclusion criteria were: (1) meeting the diagnostic criteria for pterygium [6]; (2) having no other ocular disease; (3) being able to watch and listen to audiovisual materials; (4) being scheduled for surgical treatment; and (5) providing signed informed consent.

**Figure 1:**
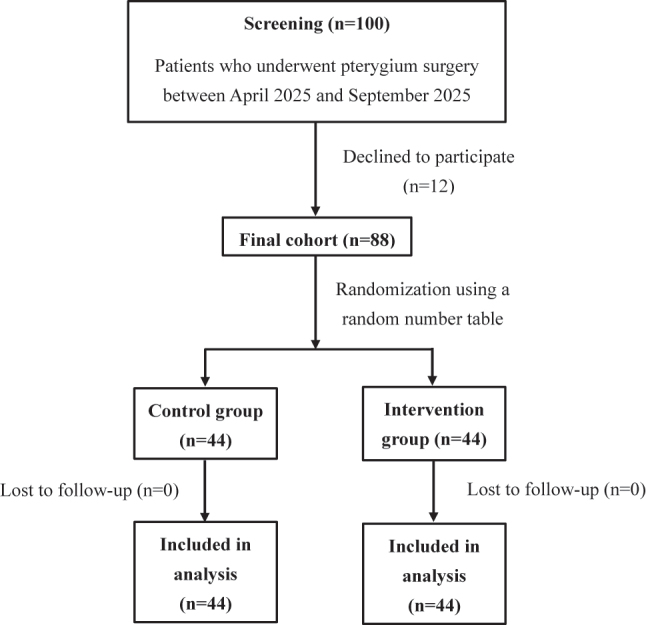
Study flow chart.

Exclusion criteria were: (1) bilateral disease; (2) history of antipsychotic drug abuse; (3) history of ocular trauma; and (4) participation in concurrent interventional studies. In the initial screening phase, 100 patients were identified for potential enrollment. Among them, 12 patients declined to participate, resulting in a final cohort of 88 participants who were included in the study. These 88 patients were then randomly allocated into a control group and an intervention group using a random number table, with 44 patients in each group. A study flow chart is shown in Figure 1.

Randomization was independently performed by researchers not involved in clinical care. The study was conducted under double-blind conditions, and no participants were excluded after randomization. All 88 patients completed the follow-up period, with no loss to follow-up.

This study was conducted with approval from the Ethics Committee of Xining No. 1 People’s Hospital (No. 2026–002). This study was conducted in accordance with the declaration of Helsinki. Written informed consent was obtained from all participants.

### Methods

The control group received routine high-quality nursing care:

(1) Preoperative care: Patients were informed of basic knowledge regarding pterygium and were made aware of the benefits of advancements in modern surgical techniques to alleviate concerns related to invasive treatment. Support was provided for the completion of ophthalmic examinations, and antibacterial eye drops were administered.
(2) Intraoperative care: Positioning focused on maintaining comfort within the limits of surgical requirements.
(3) Postoperative care: Patients were informed of the purpose of eye dressing and instructed to avoid collisions and eye rubbing. Protective glasses were required during outdoor activities. Patients were advised to instill eye drops as prescribed and avoid irritating foods.

The intervention group received the same routine high-quality nursing care supplemented with additional interventions:

(1) Multimodal health education: ① Hand hygiene education: Patients were informed of common situations requiring hand cleaning, including before meals, after using the restroom, after contact with tears, and before touching the eyes, mouth, or nose. The professional seven-step handwashing method was taught, involving washing the palms, dorsal hands, interdigital areas, and finger gaps, followed by drying with disposable paper towels. ② Preoperative education regarding disease and surgical knowledge: The causes of pterygium, such as long-term environmental irritation and aging, were explained, along with detailed information on its potential harms, including the induction of astigmatism and impairment of visual fields. Patients were informed that surgical treatment is well developed, and possible secondary injury remains within a controllable range to reduce unnecessary concerns. ③ Postoperative guidance: Patients were instructed on the correct method for eye-drop instillation. Hands were to be thoroughly cleaned under running water or with alcohol-based hand gel. Patients were instructed to look upward, gently pull down the lower eyelid using a medical cotton swab, instill the drops, and gently rotate the eyeball. Patients were advised to avoid water exposure at the surgical site and avoid unconscious eye rubbing for 1 week postoperatively. The face was to be washed using a wet towel, and bending over or lifting heavy objects that could increase intraocular pressure was avoided. Swimming, tub bathing, and night driving were prohibited for 4 weeks, and strenuous exercise was avoided for 12 weeks. Protective sunglasses or goggles were required during outdoor activities, and exposure to excessive light, wind, or dust was avoided. An eye shield was required during sleep. Patients were advised to avoid smoke, cooking fumes, irritating gases, dust, and bright light in daily life, to restrict visual load, and to keep single visual tasks to ≤30 mins. ④ Multimodal educational delivery: As most patients in the region were Tibetan, brief Tibetan-language videos were prepared for individuals with low literacy or illiteracy. In these videos, medical staff demonstrated essential actions, including eye-drop instillation, wearing an eye shield, and proper facial cleaning. Concise Tibetan-language pamphlets were provided for patients with higher educational levels, using short sentences and clear spacing. Large Tibetan characters were printed on medication boxes to indicate usage instructions, and small reminder cards listing follow-up dates were distributed. Han Chinese patients received the same educational content in Mandarin with simplified Chinese characters.
(2) Preoperative ocular fixation training: Patients assumed a supine position with relaxed muscles. A visual target was positioned approximately 30 cm directly above the dominant eye. Patients were instructed to open one eye at a time and maintain fixation on the target for 60 s. Nursing staff then provided verbal instructions to shift their gaze to the left or right, maintaining fixation for another 60 s. Training lasted 5 mins per session, was conducted 2–3 times daily, and commenced 3 days before the surgical procedure, continuing until the day of surgery.

### Outcome measures

(1) Illness uncertainty

The Mishel Uncertainty in Illness Scale–Adult (MUIS-A) was used to assess illness uncertainty before and after the intervention [7]. The scale consists of four dimensions: ambiguity (score range 13–65), complexity (7–35), lack of information (7–35), and unpredictability (5–25). The total possible score ranges from 32 to 160, and higher scores indicate higher levels of illness uncertainty.

(2) Psychological stress level

The Connor–Davidson Resilience Scale (CD-RISC) was used to assess psychological resilience before and after the intervention [8]. The scale contains three dimensions: tenacity (score range 0–52), strength (0–32), and optimism (0–16). The total possible score ranges from 0 to 100, and higher scores indicate greater psychological resilience and better capacity to cope with stress.

(3) Complications

Members of the research team recorded the incidence and percentage of complications, including ocular irritation with tearing, eye pain, infection, and dry eye. Eye pain was assessed using the numerical rating scale, a widely used method for quantifying pain intensity. The scale consists of an 11-point scale ranging from 0 to 10, on which patients select the number that best reflects their subjective pain intensity. A score of 0 indicates no pain; scores of 1–3 indicate mild pain with minimal impact on daily activities; scores of 4–6 indicate moderate pain that may interfere with sleep and daily life and may require analgesic treatment; and scores of 7–10 indicate severe pain that is difficult to tolerate and markedly affects daily activities and sleep. Pain assessments were performed on the day of surgery, postoperative day 1, and postoperative week 1.

Outcome assessors were blinded to group allocation.

### Statistical analysis

Data were processed using SPSS 27.0. Measurement data conforming to a normal distribution were expressed as mean ± standard deviation ( $\overset{®}{x}$ ± SD) and analyzed using the *t*-test. Count data were expressed as n (%) and analyzed using the chi-squared (*χ* ^*2*^) test. Statistical significance was set at p<0.05.

#### Ethical statement

This study was conducted with approval from the Ethics Committee of Xining No.1 People’s Hospital (No. 2026-002).

#### Informed consent

Informed consent was obtained from all individuals included in this study.

## Results

### General data

The general characteristics of the two groups are shown in Supplementary Table 1. Patients in the control group were 40–76 years old with a mean age of (56.78 ± 5.39) years. There were 22 males and 22 females. Based on educational background, there were 19 patients with an education qualification of junior high school or above and 25 with primary school or below.

Patients in the intervention group were 41–74 years old with a mean age of (57.31 ± 5.60) years. There were 21 males and 23 females. Based on educational background, there were 17 patients with an education qualification of junior high school or above and 27 with primary school or below.

No statistically significant differences in baseline characteristics were noted between the two groups (p>0.05).

### Comparison of illness uncertainty scores between the two groups

After the intervention, the MUIS-A scores in the intervention group were significantly lower compared with the control group (p<0.05). Lower scores reflected reduced illness uncertainty and stronger disease-coping capacity (Table 1).

**Table 1:** Comparison of illness uncertainty scores between the two groups after intervention ( $\overset{®}{x}$ ± SD, points).

Group	Sample size	Ambiguity		Complexity		Lack of information		Unpredictability	
		Pre-intervention	Post-intervention	Pre-intervention	Post-intervention	Pre-intervention	Post-intervention	Pre-intervention	Post-intervention
Intervention group	44	44.87 ± 3.35	23.70 ± 3.07	21.10 ± 2.87	13.39 ± 2.20	25.89 ± 3.12	16.68 ± 2.58	17.85 ± 2.76	11.20 ± 2.15
Control group	44	45.61 ± 3.61	27.36 ± 3.42	21.89 ± 2.64	14.48 ± 2.05	26.23 ± 2.97	17.92 ± 2.71	17.61 ± 2.85	12.35 ± 2.39
*t*		0.997	5.283	1.344	2.404	0.524	2.198	0.401	2.373
p		0.322	<0.001	0.183	0.018	0.602	0.031	0.689	0.020

### Comparison of psychological stress levels between the two groups

Following the intervention, the CD-RISC scores in the intervention group were significantly higher than those in the control group (p<0.05) (Table 2). This suggests that participants in the intervention group demonstrated improved psychological resilience, reflecting enhanced ability to adapt to and cope with psychological stressors.

**Table 2:** Comparison of psychological stress resilience scores between the two groups after intervention ( $\overset{®}{x}$ ± SD, points).

Group	Sample size	Tenacity		Strength		Optimism	
		Pre-intervention	Post-intervention	Pre-intervention	Post-intervention	Pre-intervention	Post-intervention
Intervention group	44	25.53 ± 3.81	37.68 ± 4.30	14.72 ± 2.14	23.52 ± 2.95	8.27 ± 1.10	13.25 ± 1.25
Control group	44	24.98 ± 4.03	32.70 ± 4.12	15.20 ± 2.35	20.07 ± 2.63	8.41 ± 1.03	10.61 ± 1.09
*t*		0.658	5.547	1.002	5.790	0.616	10.559
p		0.512	<0.001	0.319	<0.001	0.539	<0.001

### Comparison of complications between the two groups

Differences in the incidence of eye pain, infection, and dry eye were statistically significant between the two groups (p<0.05) (Table 3).

**Table 3:** Comparison of postoperative complications [*n* (%)].

Group	Sample size	Ocular irritation with tearing	Eye pain	Infection	Dry eye
Intervention group	44	44(100.00)	5(11.36)	0	0
Control group	44	44(100.00)	35(79.55)	6(13.64)	7(15.91)
χ²		–	41.250	4.472	5.587
p		1.000^a^	<0.001	0.034	0.018

^a^indicates Fisher’s exact test.

## Discussion

Key challenges in the care of patients undergoing pterygium surgery involve two major aspects. First, limited understanding of the disease and surgical process leads to discrepancies between cognition and behavioral responses. This often results in unconscious or habitual high-risk actions such as eye rubbing and excessive visual exertion. Second, reduced psychological tolerance commonly results in unpredictable levels of cooperation during diagnosis and treatment [9], 10]. Conventional high-quality nursing demonstrates limitations, including low information retention, insufficient individualized interaction, and the absence of behavioral pre-adaptation, which limits patients’ ability to actively comply with perioperative management. Multimodal health education supports the construction of a multidimensional cognitive foundation, whereas preoperative ocular fixation training provides behavioral simulation that reduces psychological sensitivity during surgery. The combination of these approaches enhances cognitive understanding, supports behavioral pre-adaptation, and strengthens psychological readiness, thereby addressing key perioperative barriers.

In the present study, MUIS-A scores were significantly lower in the intervention group compared with the control group. This observation aligns with previous findings indicating that multimodal health education effectively reduces preoperative anxiety [11]. Additionally, the multimodal health education in this study included culturally adapted Tibetan-language videos and concise pamphlets, enabling comprehension among patients with different educational backgrounds. Tailoring educational materials in this manner avoids misinterpretation of information and minimizes misunderstandings arising from language barriers [12]. Preoperative ocular fixation training exposed patients to intraoperative verbal instructions and eye-positioning demands in advance, thereby reducing uncertainty associated with unfamiliar surgical processes [13].

When combined, these interventions provided transparency of information, cultural adaptability, and behavioral rehearsal, ultimately reducing psychological uncertainty. Higher CD-RISC scores in the intervention group reflected improved psychological resilience. Multimodal health education supported the restructuring of disease-related cognition, and ocular fixation training familiarized patients with procedural expectations [14]. These effects provided clearer perceptual anticipation of the surgical experience and increased perceived control, which helped reduce psychological stress.

With respect to postoperative complications, the incidence of eye pain and infection was lower in the intervention group compared with the control group. Preoperative psychological stress is associated with postoperative pain perception. The intervention in this investigation likely reduced psychological stress, thereby modulating central nervous sensitivity and diminishing the perception of inflammatory pain while improving tolerance [15]. Behavioral guidance within multimodal health education, including avoidance of eye rubbing and behaviors that elevate intraocular pressure, directly prevented wound friction and excessive tension, reducing acute discomfort.

This study has several limitations. First, although baseline comparisons showed no statistically significant differences between the two groups, we acknowledge that the use of *t*-tests for post-intervention comparisons does not fully account for potential residual baseline variability. A more statistically robust approach, such as analysis of covariance, could provide improved adjustment for baseline values and increase the precision of between-group effect estimates. Future studies with larger sample sizes should consider using ANCOVA or mixed-effects models. Second, some methodological details, including allocation concealment and assessor blinding procedures, were not reported in sufficient detail, which may affect the internal validity of the study. Third, the intervention consisted of both multimodal education and eye-position training, making it difficult to distinguish the individual contribution of each component to the observed outcomes. Fourth, postoperative complications were not evaluated using standardized diagnostic thresholds. Finally, the follow-up period was relatively short, limiting the assessment of long-term efficacy and safety outcomes.

## Conclusions

The combination of multimodal health education and preoperative ocular fixation training effectively reduced illness uncertainty, enhanced psychological resilience, and lowered the incidence of postoperative complications in patients undergoing pterygium surgery. By improving cognitive understanding, promoting behavioral pre-adaptation, and enhancing psychological preparedness, this integrative approach addressed key perioperative challenges. These findings support the clinical value of structured, culturally adapted health education and behavioral rehearsal as part of comprehensive perioperative care. Further research may explore the scalability of this intervention across diverse patient populations and surgical settings.

## Supplementary Material

Supplementary Material

## References

[1] Chu WK, Choi HL, Bhat AK, Jhanji V (2020). Pterygium: new insights. Eye (Lond).

[2] Eberhart L, Aust H, Schuster M, Sturm T, Gehling M, Euteneuer F (2020). Preoperative anxiety in adults – a cross-sectional study on specific fears and risk factors. BMC Psychiatry.

[3] Tom K, Phang PT (2022). Effectiveness of the video medium to supplement preoperative patient education: a systematic review of the literature. Patient Educ Couns.

[4] Huber J, Ihrig A, Yass M, Bruckner T, Peters T, Huber CG (2013). Multimedia support for improving preoperative patient education: a randomized controlled trial using the example of radical prostatectomy. Ann Surg Oncol.

[5] Chu X, Chen F, Chen B (2024). Analysis of the effect of preoperative eye position fixation training in patients with pterygium: a randomized controlled trial. Eval Health Prof.

[6] Zhao KX, Yang PZ (2013). Ophthalmology.

[7] Mishel MH (1981). The measurement of uncertainty in illness. Nurs Res.

[8] Connor KM, Davidson JR (2003). Development of a new resilience scale: the connor-davidson resilience scale (CD-RISC). Depress Anxiety.

[9] Tan XH, Lv XL, Li CJ (2023). The impact of diversified interventions based on nursing aesthetics on postoperative recovery and ocular aesthetic satisfaction in patients undergoing pterygium excision. Chin J Appl Mech.

[10] Gao F, Zhang SJ, Du XP (2022). The impact of rapid rehabilitation nursing mode on postoperative pain, early recovery, and comfort in patients undergoing pterygium surgery. Int J Nurs.

[11] Sadeghi N, Salari N, Jalali R (2025). Effect of multimedia education on anxiety and pain in patients undergoing laparoscopic cholecystectomy: a solomon four-group randomized controlled trial. Sci Rep.

[12] Liu L, Guo CJ (2024). The impact of nursing intervention based on the concept of humanistic care on psychological stress, pain, and rehabilitation outcomes in patients with pterygium. Mod Diagn Ther.

[13] Li S, Zhang YR, Wang X (2025). The application of preoperative eye position gaze training combined with health education nursing in patients undergoing pterygium excision. Int J Nurs.

[14] Xia P, Chen YL, Huang YF (2022). The impact of rapid rehabilitation nursing on the postoperative recovery process in pterygium excision patients using corneal bandage lenses. Aerospace Med J.

[15] Yang G, Li Q, Tian M, Liu M, Zhang T, Guo W (2025). Risk factors for postoperative pain in pterygium surgery patients. J Pain Res.

