# Supplementary material for: Impact of multimodal health education and preoperative ocular fixation training on psychological stress and postoperative complications in patients undergoing pterygium surgery
Source: Open Med (Wars). 2026 Jul 7;21(1):20261480. doi: 10.1515/med-2026-1480 (PMC13340829; doi:10.1515/med-2026-1480)
Supplement: Supplementary file 1 — Supplementary Material [file j_med-2026-1480_suppl_001.docx]

Supplementary Table 1. General characteristics of the two groups

|  | Control group (n=44) | Intervention group (n=44) | t/χ² | *p*-value |
| --- | --- | --- | --- | --- |
| Age (years) | 56.78±5.39 | 57.31±5.6 | 0.30 | 0.765 |
| Sex (Male/Female), n | 22/22 | 21/23 | 0.19 | 0.663 |
| Ethnic group, n (%) |  |  | 2.37 | 0.310 |
| Han | 32 (72.7)  10 (25.0%)  2 (2.3%) | 32 (72.7)  10 (22.7%)  2 (4.5%) |  |  |
| Zang |  |  |  |  |
| Hui |  |  |  |  |
